# Nationwide Trends in Bacterial Meningitis before the Introduction of 13-Valent Pneumococcal Conjugate Vaccine—Burkina Faso, 2011–2013

**DOI:** 10.1371/journal.pone.0166384

**Published:** 2016-11-10

**Authors:** Dinanibè Kambiré, Heidi M. Soeters, Rasmata Ouédraogo-Traoré, Isaïe Medah, Lassana Sangare, Issaka Yaméogo, Guetawendé Sawadogo, Abdoul-Salam Ouédraogo, Soumeya Hema-Ouangraoua, Lesley McGee, Velusamy Srinivasan, Flavien Aké, Malika Congo-Ouédraogo, Soufian Sanou, Absatou Ky Ba, Ryan T. Novak, Chris Van Beneden, MenAfriNet Consortium

**Affiliations:** 1 Centre Hospitalier Universitaire Pédiatrique Charles de Gaulle, Ouagadougou, Burkina Faso; 2 Epidemic Intelligence Service, Centers for Disease Control and Prevention, Atlanta, Georgia, United States of America; 3 National Center for Immunization and Respiratory Diseases, Centers for Disease Control and Prevention, Atlanta, Georgia, United States of America; 4 Ministère de la Santé, Ouagadougou, Burkina Faso; 5 Centre Hospitalier Universitaire-Yalgado Ouédraogo, Ouagadougou, Burkina Faso; 6 Centre Hospitalier Universitaire Souro Sanou, Bobo-Dioulasso, Burkina Faso; 7 Centre Muraz, Bobo-Dioulasso, Burkina Faso; 8 CDC Foundation, Ouagadougou, Burkina Faso; 9 Laboratoire National de Santé Publique, Ouagadougou, Burkina Faso; Universidad Nacional de la Plata, ARGENTINA

## Abstract

**Background:**

Following introduction of *Haemophilus influenzae* type b vaccine in 2006 and serogroup A meningococcal conjugate vaccine in 2010, *Streptococcus pneumoniae* (Sp) became the leading cause of bacterial meningitis in Burkina Faso. We describe bacterial meningitis epidemiology, focusing on pneumococcal meningitis, before 13-valent pneumococcal conjugate vaccine (PCV13) introduction in the pediatric routine immunization program in October 2013.

**Methods:**

Nationwide population-based meningitis surveillance collects case-level demographic and clinical information and cerebrospinal fluid (CSF) laboratory results. Sp infections are confirmed by culture, real-time polymerase chain reaction (rt-PCR), or latex agglutination, and CSF serotyped using real-time and conventional PCR. We calculated incidence rates in cases per 100,000 persons, adjusting for age and proportion of cases with CSF tested at national reference laboratories, and case fatality ratios (CFR).

**Results:**

During 2011–2013, 1,528 pneumococcal meningitis cases were reported. Average annual adjusted incidence rates were 26.9 (<1 year), 5.4 (1–4 years), 7.2 (5–14 years), and 3.0 (≥15 years). Overall CFR was 23% and highest among children aged <1 year (32%) and adults ≥30 years (30%). Of 1,528 cases, 1,036 (68%) were serotyped: 71% were PCV13-associated serotypes, 14% were non-PCV13-associated serotypes, and 15% were non-typeable by PCR. Serotypes 1 (45%) and 12F/12A/12B/44/46 (8%) were most common. Among children aged <1 year, serotypes 5 (15%), 6A/6B (13%) and 1 (12%) predominated.

**Conclusions:**

In Burkina Faso, the highest morbidity and mortality due to pneumococcal meningitis occurred among children aged <1 year. The majority of cases were due to PCV13-associated serotypes; introduction of PCV13 should substantially decrease this burden.

## Introduction

Infection with *Streptococcus pneumoniae* is one of the leading bacterial causes of morbidity and mortality worldwide and includes life-threatening presentations such as meningitis, pneumonia and sepsis [1, 2]. *S*. *pneumoniae* is one of the primary etiologies of acute bacterial meningitis, along with *Neisseria meningitidis* and *Haemophilus influenzae* type b (Hib) [3]. In the meningitis belt of sub-Saharan Africa, stretching from Senegal to Ethiopia and including 430 million people in 26 countries [4], pneumococcal meningitis epidemiology is characterized by relatively high incidence in all age groups, high case fatality, a predominance of serotype 1 after the first five years of life, and a seasonality more typically associated with meningococcal meningitis [5–9].

Burkina Faso, a landlocked West African country with a population of approximately 19 million, is entirely located within the meningitis belt and experiences hyper-endemic rates of meningitis [10, 11]. Historically, about 90% of meningitis cases during epidemics in Burkina Faso were caused by *N*. *meningitidis* serogroup A [11] and, more recently by *N*. *meningitidis* serogroup W [5, 12]. Prior to the introduction of the serogroup A meningococcal conjugate vaccine (MenAfriVac) in 2010, national bacterial meningitis surveillance was strengthened [4, 13] through improvements in specimen collection, laboratory confirmation of bacterial etiologies, data transmission, and data management. Following the successful introduction of Hib vaccine in 2006 [14] and MenAfriVac in 2010 [4], *S*. *pneumoniae* became the primary cause of bacterial meningitis. On October 31, 2013, the government of Burkina Faso introduced the 13-valent pneumococcal conjugate vaccine (PCV13) into the routine childhood immunization program, to be administered to children at 8, 12, and 16 weeks of age.

Through the improved meningitis surveillance system and further expansion of national capacity to confirm and serotype or serogroup all major bacterial etiologies of meningitis, including *S*. *pneumoniae* [4], we provide the first nationwide population-based description of bacterial meningitis and pneumococcal meningitis trends in Burkina Faso—one of the few African countries to successfully implement case-based surveillance across the entire country. We describe nationwide case-based meningitis surveillance data from 2011 to 2013, prior to the introduction of PCV13 and in the setting of widespread use of Hib vaccine and MenAfriVac.

## Methods

### National surveillance system

Burkina Faso’s nationwide bacterial meningitis surveillance system collects case-level demographic and clinical information, as well as results of cerebrospinal fluid (CSF) examination and laboratory testing, using Integrated Disease Surveillance and Response (IDSR) [4, 15] instruments in all 63 districts. World Health Organization (WHO) case-based surveillance guidelines for the African region [16] were revised in 2015 by the MenAfriNet Consortium based upon the Burkina Faso Ministry of Health experience with successful implementation of nationwide case-based meningitis surveillance.

### Case identification

Cases were classified based upon WHO case definitions [17]. A case of suspected meningitis is defined as sudden onset of fever ≥38.5°C with one of the following signs: neck stiffness, altered consciousness, or other meningeal signs (including flaccid neck, bulging fontanel, or convulsions in young children). Probable bacterial meningitis is a suspected case with turbid, cloudy, purulent, or xanthrochromic CSF; or presence of Gram negative diplococci, Gram positive diplococci, or Gram negative bacilli on microscopic examination of CSF; or a CSF white cell count >10/mm^3^. A confirmed case of meningitis is a suspected or probable case with *S*. *pneumoniae*, *N*. *meningitidis*, or *H*. *influenzae* isolated from CSF by culture or detected in CSF by real-time polymerase chain reaction (rt-PCR) or latex agglutination.

### Laboratory methods

CSF specimens are transported from healthcare facilities to district laboratories, which conduct preliminary lab testing such as cytology, Gram stain, and latex agglutination (Pastorex, Bio-Rad). The remaining CSF is inoculated in trans-isolate media when available (stored at room temperature) and also aliquoted into a cryotube (stored at -20°C) for transportation to one of five national reference laboratories for culture and rt-PCR testing.

Once a CSF specimen reaches a reference laboratory, it is visually observed for turbidity and plated on chocolate agar. Plates are incubated at 35–37°C with 5% CO_2_ and examined up to 72 hours. Genomic DNA is extracted from 200 μl of CSF or cultures using the Qiagen DNA Mini kit (Qiagen, Valencia, CA, USA) following an initial bacterial lysis step with lysozyme (Sigma, USA) and mutanolysin (Sigma, USA) as previously described [18]. rt-PCR is used to identify *S*. *pneumoniae* through targeting the *lytA* gene using primers and probes described by Carvalho et al (2007) [18]. Similarly, the *sodC* and *hpd3* genes are targeted for *N*. *meningitidis* and *H*. *influenzae*, respectively [19]. DNA with equivocal values is diluted four-fold and ten-fold and retested.

Serotyping of *S*. *pneumoniae* positive samples was performed using a series of seven sequential triplex rt-PCR assays targeting 37 pneumococcal serotypes [20; http://www.cdc.gov/streplab/pcr.html]. Samples with Ct values of ≤30 that were non-typeable by rt-PCR were further analyzed by conventional multiplex PCR serotyping, which can detect an additional 33 serotypes [21; http://www.cdc.gov/streplab/pcr.html]. If samples were *lytA*-positive and serotype-negative both on rt-PCR and conventional PCR, they were classified as non-typeable *S*. *pneumoniae*.

### Statistical methods

We analyzed national meningitis surveillance data from January 1, 2011 to December 31, 2013. By December 31, 2013 (two months after PCV13 introduction), 31.1% of children aged 0–11 months had received one dose and 8.7% two doses of PCV13. Given that no children had received the full 3-dose series by the end of 2013 and approximately two weeks are needed to mount an effective immune response, data through December 31, 2013 was included in this pre-PCV13 analysis. Residents of countries outside of Burkina Faso were excluded from analyses.

All cases were categorized according to their date of specimen collection. Pneumococcal meningitis cases were categorized as either being caused by PCV13-associated serotypes (1, 3, 4, 5, 6A, 6B, 7F, 9V, 14, 18C, 19A, 19F, or 23F) or non-PCV13-associated serotypes. Annual incidence rates (cases per 100,000 persons) were calculated using age-stratified population estimates obtained from the *Institut National de la Statistique et de la Démographie* census. Districts that did not report case-based meningitis surveillance data were excluded from the incidence calculation denominator for that year. For suspected and probable bacterial meningitis cases, crude incidence rates were reported. For laboratory-confirmed cases, annual incidence was adjusted for the age-stratified proportion of cases with CSF tested at a national laboratory, where culture and rt-PCR were performed. Within each age strata (<1 years, 1–4 years, 5–9 years, 10–14 years, and ≥15 years), the number of cases confirmed by culture or rt-PCR for a specific pathogen was divided by the number of cases with CSF tested via culture or rt-PCR at a national laboratory; this proportion was then applied to cases lacking any test results within that age strata. We calculated the positivity of each diagnostic test among total cases confirmed as *S*. *pneumoniae* by any method, as well as sensitivity among the subset of culture-positive cases. SAS version 9.3 was used for analyses.

### Study approval

This analysis of surveillance data was approved by Burkina Faso Ministry of Health ethical committee and was determined not to require Centers for Disease Control and Prevention Institutional Review Board review.

## Results

### Data completeness and quality

**Fig 1:**
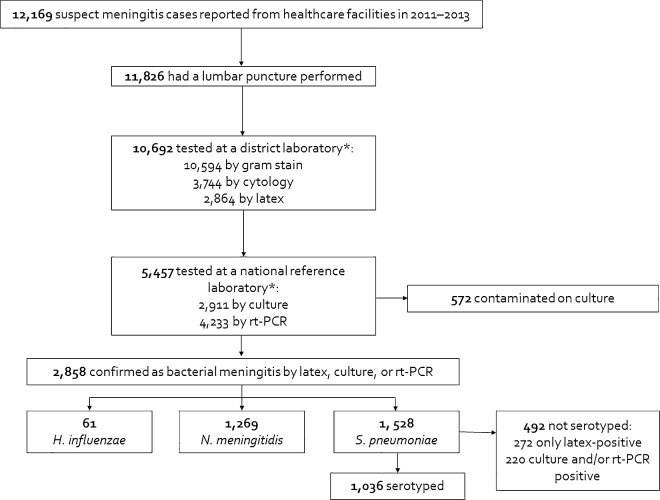
Flow diagram of the national case-based meningitis surveillance system, Burkina Faso, 2011–2013. Abbreviation: rt-PCR, real-time polymerase chain reaction. *Specimens can be tested by multiple methods.

The numbers of districts submitting data and specimens are shown in Table 1. CSF was collected from a majority (97%) of reported suspected meningitis cases; 90% of CSF specimens were tested by Gram stain at a district laboratory and 46% were subsequently tested at a national reference laboratory (Fig 1, S1 Table). Twenty-four percent of CSFs were tested by latex agglutination, 25% by culture, and 36% by rt-PCR.

**Table 1:** National meningitis data quality and completeness indicators, Burkina Faso, 2011–2013.

	2011		2012		2013		Total	
	N (%)		N (%)		N (%)		N (%)	
Districts submitting case-based surveillance data	57/63	(90)	63/63	(100)	63/63	(100)	63/63	(100)
Districts submitting CSF specimens	57/63	(90)	63/63	(100)	63/63	(100)	63/63	(100)
Suspected meningitis cases with a LP^a^	2,766	(97)	6,302	(97)	2,758	(97)	11,826	(97)
CSFs assessed for appearance	2,417	(87)	5,723	(91)	2,585	(94)	10,725	(91)
CSFs with a Gram stain^b^	2,508	(91)	5,626	(89)	2,460	(89)	10,594	(90)
CSFs tested by cytology	1217	(44)	2,473	(39)	1,271	(46)	4,961	(42)
CSFs tested at a national laboratory^c^^,^^d^	1,242	(45)	2,379	(38)	1,836	(67)	5,457	(46)
CSFs tested by latex	1,146	(41)	1,205	(19)	513	(19)	2,864	(24)
CSFs tested by culture	722	(26)	1,552	(25)	637	(23)	2,911	(25)
CSFs cultured but found to be contaminated^e^	190/722	(26)	275/1,552	(18)	107/637	(17)	572/2,911	(20)
CSFs tested by rt-PCR	1,133	(41)	1,396	(22)	1,704	(62)	4,233	(36)

Abbreviations: CSF, cerebrospinal fluid; LP, lumbar puncture; rt-PCR, real-time polymerase chain reaction.

^a^ 1,166 (10%) of CSFs were tested by latex only, 742 (6%) by culture only, 2,017 (17%) by rt-PCR only, 482 (4%) by latex and culture, 529 (4%) by latex and rt-PCR, 1,000 (8%) by culture and rt-PCR, and 687 (6%) by all three methods. 5,203 (44%) were not tested by any of the three methods.

^b^ Possible reasons why an LP may not reach a district laboratory for gram staining: difficulty with specimen transport, geographic inaccessibility of certain health centers, insufficient transport media

^c^ Defined as being tested by culture and/or rt-PCR

^d^ Possible reasons why an LP may not reach a national reference laboratory: insufficient transport media, insufficient quantity of CSF that was entirely used for Gram staining and cytology.

^e^ 447 (78%) of CSFs cultured but found to be contaminated were also tested by rt-PCR.

### National meningitis surveillance data

**Fig 2:**
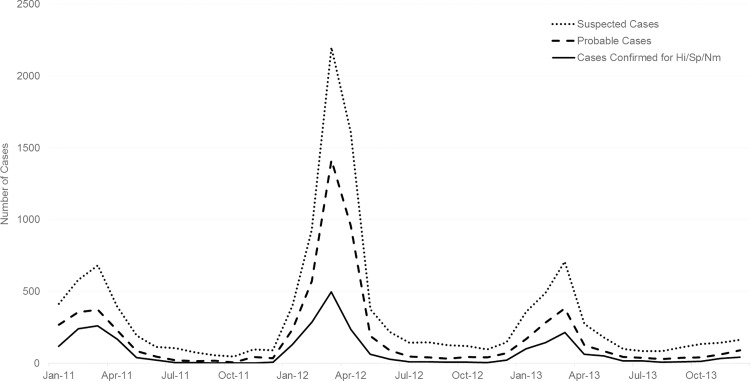
Epidemic curve of all suspected, probable, and confirmed meningitis cases by month, Burkina Faso, 2011–2013.

Over the three years of surveillance, 12,169 cases of suspected meningitis and 1,344 deaths (11%) (Table 2) were reported, corresponding to an average annual incidence of 24.7 suspected meningitis cases per 100,000 population (Table 3). The elevated number of suspected cases in 2012 was due to a nationwide outbreak of serogroup W meningococcal meningitis (Fig 2) [22]. Overall, 10,036 (82%) suspected cases occurred during meningitis season (epidemiologic weeks 1–24). A fifth of suspected cases (20%, n = 2,384) occurred among children aged <1 year, and nearly half (48%, n = 5,799) occurred in children under 5 years of age (Table 2). Few cases were reported in infants aged <1 month (n = 15) or persons aged ≥65 years (n = 149); the epidemiology in these two groups is described in S2 Table.

**Table 2:** Cases reported through national meningitis surveillance by age group and etiology, Burkina Faso, 2011–2013.

	2011		2012		2013		Total	
	N (%)		N (%)		N (%)		N (%)	
Suspected meningitis cases	2,841		6,499		2,829		12,169	
Age group^a^:								
<1 year	591	(21)	1,219	(19)	574	(20)	2,384	(20)
1 year	194	(7)	555	(9)	244	(9)	993	(8)
2–4 years	473	(17)	1,383	(21)	566	(20)	2,422	(20)
5–9 years	504	(18)	1,323	(20)	516	(18)	2,343	(19)
10–14 years	402	(14)	826	(13)	333	(12)	1,561	(13)
15–29 years	366	(13)	655	(10)	341	(12)	1,362	(11)
≥30 years	298	(11)	508	(8)	240	(9)	1,046	(9)
Reported deaths	419	(15)	590	(9)	335	(12)	1,344	(11)
Probable bacterial meningitis cases	1,488	(52)	3,727	(57)	1,377	(49)	6,592	(54)
Laboratory-confirmed meningitis cases^b^	860	(30)	1,293	(20)	705	(25)	2,858	(23)
*H*. *influenzae*^c^	26	(3)	18	(1)	17	(2)	61	(2)
Age group								
<5 years	16	(62)	13	(72)	14	(82)	43	(70)
≥5 years	10	(38)	5	(28)	3	(18)	18	(30)
Reported deaths	2	(8)	0	(0)	2	(12)	4	(7)
*N*. *meningitidis*^d^	192	(22)	813	(63)	264	(37)	1,269	(44)
Age group^e^								
<5 years	50	(26)	296	(36)	85	(32)	431	(34)
≥5 years	141	(74)	515	(64)	179	(68)	835	(66)
Reported deaths	21	(11)	62	(8)	28	(11)	111	(9)
*S*. *pneumoniae*	642	(75)	462	(36)	424	(60)	1,528	(53)
Age group:								
<1 year	104	(16)	89	(19)	83	(20)	276	(18)
1 year	19	(3)	25	(5)	21	(5)	65	(4)
2–4 years	54	(8)	46	(10)	37	(9)	137	(9)
5–9 years	134	(21)	97	(21)	81	(19)	312	(20)
10–14 years	125	(20)	81	(18)	81	(19)	287	(19)
15–29 years	114	(18)	68	(15)	71	(17)	253	(17)
≥30 years	92	(14)	56	(12)	50	(12)	198	(13)
Reported deaths	179	(28)	94	(20)	84	(20)	357	(23)

^a^ 58 cases missing age

^b^ A confirmed case of meningitis is a suspected or probable case with *S*. *pneumoniae*, *N*. *meningitidis*, or *H*. *influenzae* isolated from CSF by culture or detected in cerebrospinal fluid by real-time polymerase chain reaction or latex.

^c^ All *H*. *influenzae* cases detected in 2011–2013 were serotype b.

^d^ Serogroup was confirmed via culture or rt-PCR for 957 (75%) of 1,269 *N*. *meningitidis* cases: 751 (78%) were serogroup W, 204 (21%) were serogroup X, 1 (0.1%) was serogroup A, and 1 (0.1%) was serogroup Y.

^e^ 3 *N*. *meningitidis* cases missing age.

**Table 3:** Annual incidence (cases per 100,000 persons) of suspected, probable, and confirmed meningitis, Burkina Faso, 2011–2013.

	2011	2012	2013	Average
Suspected meningitis cases^a^	19.0	38.7	16.3	24.7
Probable bacterial meningitis cases^a^	9.2	22.2	7.9	13.1
Laboratory-confirmed meningitis cases^b^^,^^c^	9.4	16.5	5.7	10.5
*H*. *influenzae*	0.3	0.2	0.1	0.2
Age group				
<5 years	1.0	0.8	0.6	0.8
≥5 years	0.1	0.1	0.0	0.1
*N*. *meningitidis*	2.1	10.1	2.1	4.8
Age group				
<5 years	3.3	20.6	3.6	9.2
≥5 years	1.7	7.7	1.8	3.7
*S*. *pneumoniae*	7.1	6.2	3.5	5.6
Age group				
<1 years	31.3	32.9	16.5	26.9
1–4 years	6.6	6.5	3.1	5.4
5–9 years	9.2	8.1	4.0	7.1
10–14 years	9.7	7.3	4.9	7.3
≥15 years	4.0	3.1	2.0	3.0

^a^ Crude incidence

^b^ A confirmed case of meningitis is a suspected or probable case with *S*. *pneumoniae*, *N*. *meningitidis*, or *H*. *influenzae* isolated from cerebrospinal fluid (CSF) by culture or detected in CSF by real-time polymerase chain reaction or latex.

^c^ Incidence adjusted for the proportion of cases with CSF tested at a national laboratory.

Note: Incidences in the subset of high-reporting districts that provided case-based data for at least 65% of aggregately-reported cases are reported in S3 Table.

Fifty-four percent (n = 6,592) of all suspected cases met the probable case definition (Table 2, Fig 2), for an average annual incidence of 13.1 (Table 3). Among suspected cases, 2,858 (23%) were laboratory-confirmed via latex, culture, or rt-PCR as either *H*. *influenzae* (2%), *N*. *meningitidis* (44%), or *S*. *pneumoniae* (53%) (Table 2). All *H*. *influenzae* cases were serotype b; *N*. *meningitidis* serogroups are reported in a footnote of Table 2.

In 2011, 2012, and 2013, relative proportions of laboratory-confirmed bacterial meningitis cases that were caused by *S*. *pneumoniae* (75%, 36%, 60%) and *N*. *meningitidis* (22%, 63%, 37%, respectively) varied, reflecting the meningococcal outbreak in 2012, while those due to *H*. *influenzae* were rare (1–3%) (Table 2). *S*. *pneumoniae* had the highest CFR (23%), as compared to *N*. *meningitidis* (9%) and *H*. *influenzae* (7%). Overall, *S*. *pneumoniae* caused 76% of all deaths due to laboratory-confirmed bacterial meningitis. The average annual adjusted incidence for *S*. *pneumoniae*, *N*. *meningitidis*, and *H*. *influenzae*, respectively, were 5.6, 4.8, and 0.2 per 100,000 among all ages (Table 3) and 26.9, 10.8, and 2.2 per 100,000 among children aged <1 year.

Of the 1,528 confirmed pneumococcal meningitis cases reported in 2011–2013, 276 (18%) occurred among children aged <1 year, and 478 (31%) occurred in children under 5 years of age. Median age was 9 years (interquartile range: 2–16, range: 0–90). Adjusted average annual incidence was highest among children aged <1 year (26.9 per 100,000, Table 3). CFR was highest among infants and adults: 32% in children aged <1 year, 15% in children aged 1–4 years, 21% in persons aged 5–29 years, and 30% in persons aged ≥30 years. While the proportion of persons aged <1 year, 1–4 years, 5–29 years, or ≥30 years who recovered were 64%, 75%, 69%, and 66%, respectively, 12%, 14%, 16%, and 13% were recorded as ‘on treatment’ and have an unknown final outcome, and 0.0–0.6% in each age group left against medical advice.

### Pneumococcal diagnostic test results

The ability of latex, culture, and rt-PCR tests to detect *S*. *pneumoniae* varied (Table 4). Overall, 642 (84.7%) of 758 laboratory-confirmed (using any of the three diagnostic methods) pneumococcal meningitis cases tested using latex agglutination were positive for *S*. *pneumoniae*, and 217 (41.1%) of 528 tested by culture grew *S*. *pneumoniae*. The majority of pneumococcal meningitis cases were confirmed using rt-PCR: 1,210 (96.3%) of 1,256 pneumococcal cases tested by rt-PCR were positive for *S*. *pneumoniae*. Using culture as the gold standard, the sensitivities of latex agglutination and rt-PCR were 90.1% and 95.5%, respectively. Although testing practices varied by year, sensitivity of each of the three testing methods remained consistent.

**Table 4:** *S*. *pneumoniae* diagnostic test results among confirmed^a^ pneumococcal meningitis cases, Burkina Faso, 2011–2013.

Diagnostic testing method	2011		2012		2013		Total	
	**No. positive for *S*. *pneumoniae* using method(s) (%)**							
Latex only	151	(23.5)	83	(18.0)	38	(9.0)	272	(17.8)
Culture only	8	(1.3)	18	(3.9)	1	(0.2)	27	(1.8)
rt-PCR only	216	(33.6)	257	(55.6)	305	(71.9)	778	(50.9)
Latex and culture only	9	(1.4)	8	(1.7)	2	(0.5)	19	(1.2)
Latex and rt-PCR only	158	(24.6)	62	(13.4)	41	(9.7)	261	(17.1)
Culture and rt-PCR	34	(5.3)	14	(3.0)	33	(7.8)	81	(5.3)
Latex, culture and rt-PCR	66	(10.3)	20	(4.3)	4	(0.9)	90	(5.9)
	**No. positive / No. of confirmed^a^ cases tested using method (%)**							
Latex	384/453	(84.8)	173/201	(86.1)	85/104	(81.7)	642/758	(84.7)
Culture	117/271	(43.2)	60/148	(40.5)	40/109	(36.7)	217/528	(41.1)
rt-PCR	474/504	(94.0)	353/360	(98.1)	383/392	(97.7)	1,210/1,256	(96.3)
	**Sensitivity using culture as the gold standard**							
	**No. positive / No. of culture-positive cases tested using method (%)**							
Latex	75/79	(94.9)	28/34	(82.4)	6/8	(75.0)	109/121	(90.1)
rt-PCR	100/105	(95.2)	34/37	(91.9)	37/37	(100.0)	171/179	(95.5)
**Total positive for *S*. *pneumoniae***	**642**		**462**		**424**		**1,528**	

Abbreviations: rt-PCR, real-time polymerase chain reaction.

^a^ *S*. *pneumoniae* isolated from cerebrospinal fluid (CSF) by culture or detected in CSF by rt-PCR or latex. Not all specimens were tested via all three methods.

### Pneumococcal serotype distribution

Of the 1,528 pneumococcal meningitis cases, 1,036 (68%) were serotyped (Table 5 and S4 Table). Eighteen percent (n = 272) of cases were only positive via latex agglutination, so could not be serotyped; 220 culture- or rt-PCR-positive cases did not have a specimen or isolate available for serotyping (107 from 2011, 53 from 2012, and 60 from 2013) due to lack of isolate storage, inability to locate isolates, or insufficient DNA available for serotyping. Overall, 737 (71%) were PCV13-associated serotypes, 141 (14%) were non-PCV13-associated serotypes, and 158 (15%) were non-typeable. The predominant serotypes by age group were serotypes 5 (15%), 6A/6B (13%) and 1 (12%) among children aged <1 year; serotypes 1 (23%) and 6A/6B (11%) among ages 1–4 years; and serotypes 1 (57%) and 12F/12A/12B/44/46 (8%) in persons aged ≥5 years (Table 5). Among children aged <5 years, 66% of cases were due to PCV13-associated serotypes. Overall, serotype 1 was responsible for 63% of PCV13-associated cases.

**Table 5:** Distribution of pneumococcal serotypes by age, Burkina Faso, 2011–2013.

Pneumococcal serotype	<1 year,N (%)		1–4 years,N (%)		≥5 years,N (%)		Total,N (%)	
***PCV13-associated***	***124***	***(65)***	***83***	***(67)***	***530***	***(74)***	***737***	***(71)***
1	22	(12)	28	(23)	414	(57)	464	(45)
3	0	(0)	1	(1)	4	(1)	5	(0.5)
4	2	(1)	1	(1)	7	(1)	10	(1)
5	28	(15)	8	(6)	35	(5)	71	(7)
6A/6B	24	(13)	14	(11)	8	(1)	46	(4)
7F/7A	4	(2)	4	(3)	8	(1)	16	(2)
9V/9A	2	(1)	0	(0)	1	(0.1)	3	(0.3)
14	14	(7)	6	(5)	14	(2)	34	(3)
18C/18F/18B/18A	8	(4)	4	(3)	8	(1)	20	(2)
19A	4	(2)	1	(1)	4	(1)	9	(1)
19F	2	(1)	5	(4)	4	(1)	11	(1)
23F	14	(7)	11	(9)	23	(3)	48	(5)
***Non-PCV13-associated***	***37***	***(19)***	***15***	***(12)***	***89***	***(12)***	***141***	***(14)***
2	13	(7)	0	(0)	4	(1)	17	(2)
7C/7B/40	0	(0)	0	(0)	0	(0)	0	(0)
8	0	(0)	0	(0)	2	(0.3)	2	(0.2)
9N/9L	2	(1)	1	(1)	1	(0.2)	4	(0.4)
10F/10C/33C	1	(1)	0	(0)	0	(0)	1	(0.1)
11A/11D	0	(0)	0	(0)	1	(0.1)	1	(0.1)
12F/12A/12B/44/46	18	(9)	7	(6)	57	(8)	82	(8)
13	0	(0)	0	(0)	1	(0.1)	1	(0.1)
15B/15C	0	(0)	0	(0)	5	(1)	5	(0.5)
16F	1	(1)	0	(0)	3	(0.4)	4	(0.4)
21	0	(0)	2	(2)	0	(0)	2	(0.2)
22F/22A	0	(0)	0	(0)	2	(0.3)	2	(0.2)
23B	0	(0)	1	(1)	0	(0)	1	(0.1)
24F/24A/24B	0	(0)	1	(1)	1	(0.1)	2	(0.2)
25F/25A/38	0	(0)	3	(2)	10	(1)	13	(1)
33F/33A/37	1	(1)	0	(0)	0	(0)	1	(0.1)
34	0	(0)	0	(0)	1	(0.1)	1	(0.1)
35B	1	(1)	0	(0)	1	(0.1)	2	(0.2)
***Non-typeable***	***30***	***(16)***	***26***	***(21)***	***102***	***(14)***	***158***	***(15)***
**Total serotyped**	**191**	**(69)**	**124**	**(61)**	**721**	**(69)**	**1,036**	**(68)**
**Missing serotype**^**a**^	**85**	**(31)**	**78**	**(39)**	**329**	**(31)**	**492**	**(32)**
**Total**	**276**		**202**		**1,050**		**1,528**	

^a^ 272 cases were positive only via latex agglutination and could not be serotyped: 151 from 2011, 83 from 2012, and 38 from 2013. Serotype results were unavailable for 220 culture- and/or rt-PCR-positive cases: 107 from 2011, 53 from 2012, and 60 from 2013.

### Temporal trends

**Fig 3:**
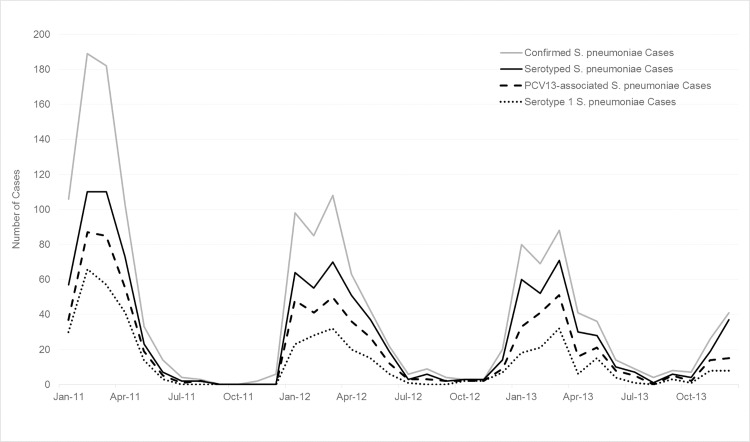
Epidemic curve of confirmed* pneumococcal meningitis cases, serotyped pneumococcal meningitis cases, PCV13-associated pneumococcal meningitis cases, and serotype 1 pneumococcal meningitis cases by month, Burkina Faso, 2011–2013. *S. *pneumoniae* isolated from cerebrospinal fluid (CSF) by culture or detected in CSF by real-time polymerase chain reaction or latex.

**Fig 4:**
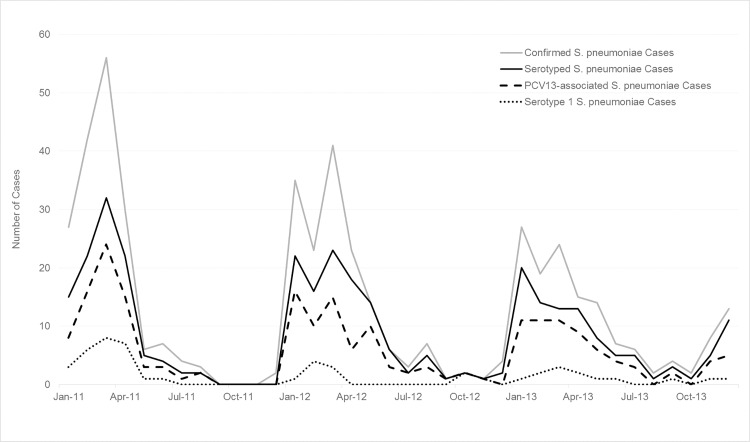
Epidemic curve of confirmed* pneumococcal meningitis cases, serotyped pneumococcal meningitis cases, PCV13-associated pneumococcal meningitis cases, and serotype 1 pneumococcal meningitis cases among children aged <5 years, by month, Burkina Faso, 2011–2013. *S. *pneumoniae* isolated from cerebrospinal fluid (CSF) by culture or detected in CSF by real-time polymerase chain reaction or latex.

Epidemic curves of the various case definitions of meningitis and of pneumococcal meningitis cases (all ages and those aged <5 years) by month of specimen collection are shown in Figs 2–4. Monthly trends followed the seasonality of meningitis epidemics typically observed in the meningitis belt. The marked peak of meningococcal meningitis cases during the serogroup W outbreak in 2012 is reflected in the increase in suspect, probable, and lab-confirmed cases in Fig 2. In comparison, the seasonal peaks of pneumococcal meningitis cases showed less variation by year with the greatest activity in 2011 and successively smaller peaks in 2012 and 2013 (Figs 3 and 4). Seasonality of PCV13-associated cases and serotype 1 cases was similar to that of overall pneumococcal meningitis.

## Discussion

We report the first nationwide population-based description of bacterial meningitis, and specifically pneumococcal meningitis, in Burkina Faso following widespread use of Hib and serogroup A meningococcal conjugate vaccines. Pneumococcal meningitis was responsible for the majority (53%) of laboratory-confirmed bacterial meningitis cases overall, and was characterized by a higher case fatality (23%) than *N*. *meningitidis* (9%) and *H*. *influenzae* (7%). A third of pneumococcal meningitis cases in Burkina Faso were in children aged <5 years, with highest morbidity and mortality among children aged <1 year. Overall, serotype 1 was responsible for 63% of all PCV13-associated infections and the proportion of pneumococcal infections due to serotype 1 increased with age. Pneumococcal meningitis demonstrated a seasonality typically associated with meningococcal meningitis, with 75% to 97% of cases occurring during meningitis season each year.

In this study, *S*. *pneumoniae* was responsible for the majority of laboratory-confirmed bacterial meningitis during non-meningococcal epidemic years– 2 of the 3 years we evaluated. Most previous studies in Burkina Faso present data collected prior to the mass MenAfriVac vaccination campaign in 2010. Therefore, with serogroup A meningococcus still circulating, these earlier studies reported a relatively low proportion of bacterial meningitis that was pneumococcal, ranging from 14% to 44% [5–7, 23].

These results of national meningitis surveillance—a high case fatality of pneumococcal meningitis, a predominance of serotype 1 after the first five years of life, and seasonality—reaffirmed findings of previous pneumococcal meningitis studies that were based on surveillance in 3 to 4 of the 63 districts in Burkina Faso [5–7, 9]. However, our adjusted average annual pneumococcal meningitis incidence of 5.6 cases per 100,000 in this national surveillance system was lower than many previously reported in the region (range: 9 to 17) [5–7, 24], although we found children aged <1 year had an incidence nearly nine times that observed among other ages, which is consistent with previous reports [7, 8]. Our reported pneumococcal meningitis incidence in children aged <5 years (10.1 per 100,000) was also lower than the estimated incidence modelled by O’Brien *et al* for the Africa region in 2000 (38 per 100,000, uncertainty range: 11–48) [1]. Our reported incidences may be lower for a number of reasons: 1) while previous studies report sentinel surveillance incidences or use sentinel incidences to estimate national incidences, we report full nationwide incidence; 2) this analysis describes cases detected through routine clinical care and reported through routine channels as part of nationwide passive surveillance, as opposed to active, sentinel surveillance supported by special research studies and additional infrastructure; some cases were likely undiagnosed or unreported; 3) sentinel surveillance incidence rates based on persons seeking care at a referral hospital may be inaccurate if persons not included in the surveillance catchment area (denominator) seek care for meningitis and are included, or if cases seek care outside the catchment area; 4) sentinel surveillance sites or special research studies may be purposely placed in areas with high meningitis incidence or urban areas with higher population density and therefore higher potential for transmission; and 5) this analysis uses recent data from 2011 to 2013, perhaps reflecting true decreases in incidence since previous studies were completed. Although this national surveillance system likely results in underreporting, the methods used here are more sustainable for countries in the region and enable analysis of disease trends.

We reported a CFR of 23% for pneumococcal meningitis in cases of all ages, lower than all previously-reported estimates (40%-47%) in the region [5–8, 23, 24]. Across all reports the mortality was highest among children aged <1 year. Our CFR may be lower due to incomplete capture of outcomes nationwide; many patients were reported as being ‘on treatment’ and their final outcome was not subsequently documented by national surveillance. However, incomplete mortality reporting would equally occur with all pathogens, and *S*. *pneumoniae* was still responsible for 76% of bacterial meningitis deaths in Burkina Faso. This highlights the high comparative burden of pneumococcal meningitis mortality, which has been noted previously [5–7]. We could not assess the full burden or serotype distribution of pneumococcal infections as Burkina Faso does not conduct surveillance for other pneumococcal syndromes such as pneumonia or sepsis. Also, there was no available data on disease-related sequelae other than deaths, which can affect as many as half of children with pneumococcal meningitis [25–27].

Serotype 1 continues to predominate in this region, causing 45% of pneumococcal meningitis in all age groups and 57% of cases aged ≥5 years. This overall proportion is nearly identical to that reported previously [6, 9, 28]. In young children, serotypes 1, 5, and 6A/6B caused 40% of serotyped cases; we found fewer cases caused by serotypes 2, 3, 14, 2, and 18 than reported previously [7, 8, 29]. Among children aged <5 years, 66% of disease was covered by serotypes included in PCV13, consistent with estimates (53–67%) in previous reports [7, 9], indicating that PCV13 introduction has potential for substantial impact on disease burden among children. PCV13-associated serotypes caused 74% of pneumococcal meningitis in persons aged ≥5 years, indicating that widespread use of PCV13 among infants could have a major impact on meningitis among older age groups through indirect protection. However, given that most cases due to PCV13-associated serotypes were in fact caused by serotype 1, a serotype rarely found in colonization studies [30], the effect of PCV13 on herd immunity among non-vaccinated persons in Burkina Faso remains to be seen. We found a high proportion (15%) of non-typeables, despite using both rt-PCR and conventional PCR methods to serotype all pneumococcal isolates and specimens. This is consistent with previous studies in the region [9], and may reflect circulating serotypes that are not covered by existing methods.

The national case-based meningitis surveillance system in Burkina Faso is continually improving. All districts are now submitting CSF specimens and the proportion of CSFs tested by culture or rt-PCR at a national laboratory increased from 45% in 2011 to 67% by 2013. We adjusted incidence rates to partially account for this under-testing. However, challenges remain, particularly with increasing timely specimen transport to national laboratories and ensuring that the proper transport media and containers are available where and when they are needed. As case ascertainment and outcome reporting continue to improve, this national surveillance system produces robust nationwide estimates that can be considered a lower bound for estimates of incidence and CFR.

In our study, the positivity of culture for diagnosis of pneumococcal meningitis was low (41%). CSF culture is considered the gold standard for diagnosis of bacterial meningitis and is positive in 80–90% of patients with acute community-acquired bacterial meningitis before the start of treatment [31]. Our low culture positivity is likely due to a number of reasons including 1) prior antibiotic use; 2) delayed culture of CSF specimen due to lack of culture capacity at regional laboratories; 3) lack of availability of blood agar plates for optimal recovery of pneumococci, and 4) a high proportion of contaminated cultures (20%). The strengthening of the national bacterial meningitis surveillance system to include capacity for rt-PCR testing at national laboratories significantly increased pneumococcal meningitis case confirmation and also provided a platform for pneumococcal serotyping.

This analysis is the first nationwide population-based report of pneumococcal meningitis epidemiology in Burkina Faso and the first to include routine serotyping of pneumococcal specimens throughout the entire country. Serotyping all pneumococcal meningitis specimens as part of the national surveillance system is essential to understanding the epidemiology and evaluating pneumococcal vaccination efforts. This data analysis serves as a baseline against which PCV13 impact can be measured. Introduction of PCV13 should decrease the burden of pneumococcal meningitis, as most cases were due to vaccine-associated serotypes. Additionally, widespread use of PCV13 among infants could have a major impact on pneumococcal disease among older children and adults if the infant routine immunization program induces indirect protection against serotype 1 and other PCV13-associated serotypes.

## Supporting Information

S1 File Dataset. (XLSX)

S1 Table Comparison of suspected meningitis cases tested vs. not tested at a national reference lab, Burkina Faso, 2011–2013. (PDF)

S2 Table Bacterial meningitis epidemiology in infants aged <1 month and persons aged ≥65 years, Burkina Faso, 2011–2013. (PDF)

S3 Table Re-calculated incidences in the subset of high-reporting districts that provided case-based data for at least 65% of aggregately-reported cases each year. (PDF)

S4 Table Distribution of pneumococcal serotypes, Burkina Faso, 2011–2013. (PDF)
