# Supplementary material for: Nationwide Trends in Bacterial Meningitis before the Introduction of 13-Valent Pneumococcal Conjugate Vaccine—Burkina Faso, 2011–2013
Source: PLoS One. 2016 Nov 10;11(11):e0166384. doi: 10.1371/journal.pone.0166384 (PMC5104358; doi:10.1371/journal.pone.0166384)
Supplement: S1 Table — (PDF) [file pone.0166384.s002.pdf]

S1 Table. Comparison of suspected meningitis cases tested[a] vs. not tested at a national reference lab, Burkina Faso, 2011–2013

	2011				2012				2013				Total			
	Tested N(%)		Not tested N(%)		Tested N(%)		Not tested N(%)		Tested N(%)		Not tested N(%)		Tested N(%)		Not tested N(%)	
Suspect cases	1,242	(44)	1,599	(56)	2,379	(37)	4,120	(63)	1,836	(65)	993	(35)	5,457	(45)	6,712	(55)
Age group[b]:																
<1 years	240	(19)	351	(22)	408	(17)	811	(20)	384	(21)	190	(19)	1,032	(19)	1,352	(20)
1-4 years	234	(19)	433	(27)	679	(29)	1,259	(31)	518	(28)	292	(30)	1,431	(26)	1,984	(30)
5-9 years	237	(19)	267	(17)	515	(22)	808	(20)	357	(19)	159	(16)	1,109	(20)	1,234	(19)
10-14 years	202	(16)	200	(13)	345	(15)	481	(12)	223	(12)	110	(11)	770	(14)	791	(12)
≥15 years	326	(26)	338	(21)	424	(18)	739	(18)	351	(19)	230	(23)	1,101	(20)	1,307	(20)
Gram stain:																
Not done	90	(7)	243	(15)	89	(4)	784	(19)	116	(6)	253	(25)	295	(5)	1,280	(19)
Done	1,152	(93)	1,356	(85)	2,290	(96)	3,336	(81)	1,720	(94)	740	(75)	5,162	(95)	5,432	(81)
BGN	20	(2)	33	(2)	26	(1)	52	(1)	21	(1)	10	(1)	67	(1)	95	(1)
BGP	1	(0.1)	9	(1)	6	(0.3)	20	(0.5)	4	(0.2)	9	(1)	11	(0.2)	38	(1)
DGN	89	(7)	108	(7)	553	(23)	838	(20)	183	(10)	65	(7)	825	(15)	1,011	(15)
DGP	378	(30)	264	(17)	315	(13)	363	(9)	296	(16)	128	(13)	989	(18)	755	(11)
Negative	664	(53)	942	(59)	1,390	(58)	2,063	(50)	1,216	(66)	528	(53)	3,270	(60)	3,533	(53)
Probable case[c]:																
Yes	800	(64)	688	(43)	1,459	(61)	2,268	(55)	953	(52)	424	(43)	3,212	(59)	3,380	(50)
No	442	(36)	911	(57)	920	(39)	1,852	(45)	883	(48)	569	(57)	2,245	(41)	3,332	(50)
Reported death																
Yes	199	(16)	220	(14)	184	(7)	406	(10)	190	(10)	145	(15)	573	(11)	771	(11)
No	1,043	(84)	1,379	(86)	2,195	(92)	3,714	(90)	1,646	(90)	848	(85)	4,884	(90)	5,941	(89)

Abbreviations: BGN, gram negative bacilli; BGP, gram positive bacilli; CSF, cerebrospinal fluid; DGN, gram negative diplococci; DGP, gram positive diplococci.

[a] A suspected meningitis case was defined as being tested at a national reference lab if it was tested by culture and/or real-time polymerase chain reaction.

[b] 58 cases missing age

[c] Probable bacterial meningitis is a suspected case with turbid, cloudy, purulent, or xanthrochromic CSF; or presence of DGN, DGP, or BGN on microscopic examination of CSF; or a CSF white cell count >10/mm^3.
