# Supplementary material for: Nationwide Trends in Bacterial Meningitis before the Introduction of 13-Valent Pneumococcal Conjugate Vaccine—Burkina Faso, 2011–2013
Source: PLoS One. 2016 Nov 10;11(11):e0166384. doi: 10.1371/journal.pone.0166384 (PMC5104358; doi:10.1371/journal.pone.0166384)
Supplement: S2 Table — (PDF) [file pone.0166384.s003.pdf]

S2 Table. Bacterial meningitis epidemiology in infants aged <1 month and persons aged ≥65 years, Burkina Faso, 2011–2013

	<1 month N (%)		≥65 years N (%)	
Suspected meningitis cases	15		149	
Reported deaths	1	(7)	36	(24)
Probable bacterial meningitis cases	7	(47)	78	(52)
Laboratory-confirmed meningitis cases[a]	4	(27)	34	(23)
H. influenzae	0	(0)	0	(0)
N. meningitidis	1	(25)	5	(15)
Reported deaths	0	(0)	0	(0)
Meningococcal serogroup				
W	1	(100)	2	(67)
X	0	(0)	1	(33)
Missing serogroup	0		2	
S. pneumoniae	3	(75)	29	(85)
Reported deaths	1	(33)	14	(48)
Pneumococcal serotype				
1	1	(100)	6	(30)
4	0	(0)	1	(5)
7F/7A	0	(0)	2	(10)
12F/12A/12B/44/46	0	(0)	4	(20)
19A	0	(0)	1	(5)
23F	0	(0)	1	(5)
Non-typeable	0	(0)	5	(25)
Missing serotype	2		9	

[a] A confirmed case of meningitis is a suspected or probable case with *S. pneumoniae*, *N. meningitidis*, or *H. influenzae* isolated from CSF by culture or detected in cerebrospinal fluid by real-time polymerase chain reaction or latex agglutination.
