# Supplementary material for: Nationwide Trends in Bacterial Meningitis before the Introduction of 13-Valent Pneumococcal Conjugate Vaccine—Burkina Faso, 2011–2013
Source: PLoS One. 2016 Nov 10;11(11):e0166384. doi: 10.1371/journal.pone.0166384 (PMC5104358; doi:10.1371/journal.pone.0166384)
Supplement: S3 Table — (PDF) [file pone.0166384.s004.pdf]

S3 Table. Re-calculated incidences in the subset of high-reporting districts[a] that provided case-based data for at least 65% of aggregately-reported cases each year

	2011	2012	2013	Average
Suspected meningitis cases[b]	23.9	40.0	17.0	27.0
Probable bacterial meningitis cases[b]	12.5	23.1	8.3	14.6
Laboratory-confirmed meningitis cases[c,d]	11.9	14.0	4.2	10.0
H. influenzae	0.3	0.2	0.1	0.2
Age group				
<5 years	1.1	0.7	0.4	0.7
≥5 years	0.2	0.1	0.0	0.1
N. meningitidis	2.8	8.7	1.6	4.4
Age group				
<5 years	4.5	16.7	2.7	8.0
≥5 years	2.4	6.8	1.3	3.5
S. pneumoniae	8.7	5.1	2.6	5.5
Age group				
<1 years	42.3	24.0	12.1	26.1
1-4 years	9.4	5.1	2.4	5.6
5-9 years	11.6	7.1	3.1	7.2
10-14 years	11.2	6.7	3.6	7.2
≥15 years	4.4	2.6	1.4	2.8

[a] Of 63 districts, 39 were high-reporting in 2011, 59 in 2012, and 62 in 2013.

[b] Crude incidence

[c] A confirmed case of meningitis is a suspected or probable case with *S. pneumoniae*, *N. meningitidis*, or *H. influenzae* isolated from cerebrospinal fluid (CSF) by culture or detected in CSF by real-time polymerase chain reaction or latex.

[d] Incidence adjusted for the proportion of cases with CSF tested at a national laboratory.
