# Supplementary material for: Nationwide Trends in Bacterial Meningitis before the Introduction of 13-Valent Pneumococcal Conjugate Vaccine—Burkina Faso, 2011–2013
Source: PLoS One. 2016 Nov 10;11(11):e0166384. doi: 10.1371/journal.pone.0166384 (PMC5104358; doi:10.1371/journal.pone.0166384)
Supplement: S4 Table — (PDF) [file pone.0166384.s005.pdf]

S4 Table. Distribution of pneumococcal serotypes, Burkina Faso, 2011–2013

Pneumococcal serotype	2011 N	(%)	2012 N	(%)	2013 N	(%)	Total N	(%)
PCV13-associated	***291***	***(76)***	***234***	***(72)***	***212***	***(65)***	***737***	***(71)***
1	211	(55)	135	(41)	118	(36)	464	(45)
3	1	(0.3)	2	(1)	2	(1)	5	(0.5)
4	5	(1)	2	(1)	3	(1)	10	(1)
5	35	(9)	21	(6)	15	(5)	71	(7)
6A/6B	8	(2)	19	(6)	19	(6)	46	(4)
7F/7A	4	(1)	5	(2)	7	(2)	16	(2)
9V/9A	0	(0)	2	(1)	1	(0.3)	3	(0.3)
14	16	(4)	7	(2)	11	(3)	34	(3)
18C/18F/18B/18A	2	(1)	12	(4)	6	(2)	20	(2)
19A	1	(0.3)	5	(2)	3	(1)	9	(1)
19F	2	(1)	3	(1)	6	(2)	11	(1)
23F	6	(2)	21	(6)	21	(6)	48	(5)
Non-PCV13-associated	***30***	***(8)***	***49***	***(15)***	***62***	***(19)***	***141***	***(14)***
2	3	(1)	8	(2)	6	(2)	17	(2)
7C/7B/40	0	(0)	0	(0)	0	(0)	0	(0)
8	0	(0)	0	(0)	2	(1)	2	(0.2)
9N/9L	0	(0)	2	(1)	2	(1)	4	(0.4)
10F/10C/33C	0	(0)	0	(0)	1	(0.3)	1	(0.1)
11A/11D	1	(0.3)	0	(0)	0	(0)	1	(0.1)
12F/12A/12B/44/46	20	(5)	26	(8)	36	(11)	82	(8)
13	0	(0)	1	(0.3)	0	(0)	1	(0.1)
15B/15C	3	(1)	0	(0)	2	(1)	5	(0.5)
16F	0	(0)	2	(1)	2	(1)	4	(0.4)
21	1	(0.3)	1	(0.3)	0	(0)	2	(0.2)
22F/22A	1	(0.3)	1	(0.3)	0	(0)	2	(0.2)
23B	0	(0)	0	(0)	1	(0.3)	1	(0.1)
24F/24A/24B	0	(0)	0	(0)	2	(1)	2	(0.2)
25F/25A/38	1	(0.3)	6	(2)	6	(2)	13	(1)
33F/33A/37	0	(0)	1	(0.3)	0	(0)	1	(0.1)
34	0	(0)	0	(0)	1	(0.3)	1	(0.1)
35B	0	(0)	1	(0.3)	1	(0.3)	2	(0.2)
Non-typeable	***63***	***(16)***	***43***	***(13)***	***52***	***(16)***	***158***	***(15)***
**Total serotyped**	**384**	**(60)**	**326**	**(71)**	**326**	**(77)**	**1,036**	**(68)**
**Missing serotype[a]**	**258**	**(40)**	**136**	**(29)**	**98**	**(23)**	**492**	**(32)**
**Total**	**642**		**462**		**424**		**1,528**	

[a] 272 cases were only positive via latex agglutination and could not be serotyped: 151 from 2011, 83 from 2012, and 38 from 2013. Serotype results were unavailable for 220 culture- and/or rt-PCR-positive cases: 107 from 2011, 53 from 2012, and 60 from 2013.
